# Bioinformatics calls the school: Use of smartphones to introduce Python for bioinformatics in high schools

**DOI:** 10.1371/journal.pcbi.1006473

**Published:** 2019-02-14

**Authors:** Ana Julia Velez Rueda, Guillermo I. Benítez, Julia Marchetti, Marcia Anahí Hasenahuer, María Silvina Fornasari, Nicolas Palopoli, Gustavo Parisi

**Affiliations:** Departamento de Ciencia y Tecnología, Universidad Nacional de Quilmes - CONICET, Bernal, Buenos Aires, Argentina; Genome Quebec, CANADA

## Abstract

The dynamic nature of technological developments invites us to rethink the learning spaces. In this context, science education can be enriched by the contribution of new computational resources, making the educational process more up-to-date, challenging, and attractive. Bioinformatics is a key interdisciplinary field, contributing to the understanding of biological processes that is often underrated in secondary schools. As a useful resource in learning activities, bioinformatics could help in engaging students to integrate multiple fields of knowledge (logical-mathematical, biological, computational, etc.) and generate an enriched and long-lasting learning environment. Here, we report our recent project in which high school students learned basic concepts of programming applied to solving biological problems. The students were taught the Python syntax, and they coded simple tools to answer biological questions using resources at hand. Notably, these were built mostly on the students’ own smartphones, which proved to be capable, readily available, and relevant complementary tools for teaching. This project resulted in an empowering and inclusive experience that challenged differences in social background and technological accessibility.

## Author summary

Bioinformatics is an interdisciplinary activity that complements and connects several fields with biology and can also be used as an educational tool for science. During 2017, the Structural Bioinformatics Group at National University of Quilmes in Buenos Aires, Argentina, worked together with public and private schools to promote the usage of bioinformatics towards a better understanding of biology. We performed short biology-oriented programming workshops using Python, aimed at students in different schools, who were later invited to participate in a specially organized and challenging bioinformatics contest (http://ufq.unq.edu.ar/sbg/education/index.html). The choice of computational tools, with a major role of smartphone applications, made the teaching–learning process easier, dynamic, and accessible. This experience allowed us to build bridges with the participating schools and develop a great commitment toward expanding the project in the near future. The great interest shown by educational communities and the positive responses of students reinforce the idea of bioinformatics as a plausible tool for the learning–teaching of biology.

## Background and motivation

There have been many difficulties in the last years for the incorporation of information and communication technologies (ICTs) as a strategy for teaching and learning biology in the classroom [1]. In the context of an increased public access to ICTs [2], the new challenge is to incorporate and benefit from them, to encouraging students to perform complex operations, and to take full advantage of these resources for significant learning in multiple areas [3]. As an interdisciplinary field, bioinformatics provides the ideal means to advance in these goals. Bioinformatics researchers employ computational techniques to generate and improve biological and biochemical knowledge [4], contributing in many ways to clinical, academic, and industrial development [5–8] while generating useful tools for educational usage [9,10]. For several reasons and despite its many potential benefits, bioinformatics is still waiting to be incorporated into educational curriculums in many countries.

The usage of integrated technologies in basic education represents a challenge for teachers, who must transform their daily practices in order to inspire real intellectual curiosity in students [11] [12]. But the challenge is not only educational, because teachers need to deal with the sociological and economic aspects influencing the school and its students [13]. For example, the available resources depend on the country’s public policies, the region and its development status, the current economic context, whether the schools are private or public, etc. [14]. In this general context, it is hard to ensure the computational resources required for teaching bioinformatics.

In countries like Argentina, for example, in which new technologies are often accessible to students (a recent study by the National Institute of Statistics and Censuses [INDEC] [15]shows that although only 67% of the Argentinian households own a computer, 89% of these families have at least a mobile phone), there are certainly other difficulties for the integration of ICTs in educational practices that are commonly hard to overcome. A recent UNICEF report [16] indicates that although 89% of Argentinian schools own at least one computer accessible to staff and students, and almost all their teachers consider implementing computers for pedagogical uses, only half of these teachers actually incorporate computers in the classroom. The same study shows that mobile phones constitute a particular case for a tool that is not provided by schools but that students can routinely access. It is interesting to notice that even when studies around the world show the big potential of smartphones as pedagogical tools [17,18] [19,20], just 44% of Argentine teachers consider these as instruments to incorporate in their classroom, with only a quarter of them currently allowing educational uses of mobile phones.

In this work, we summarize a one-year-long experience working with different public and private Argentinian schools to promote the usage of bioinformatics as a teaching–learning tool for biology. This project, driven by the Structural Bioinformatics Group at National University of Quilmes in Buenos Aires, aimed at providing alternative ways of teaching biology by validating and rediscovering technology elements of daily use as tools for scientific development. Almost a hundred students learned basic concepts of programming in order to build their own programs to answer biological questions. They ran free implementations of the Python language, mostly on their own smartphones. This project resulted in a rich experience that empowered students of different social backgrounds with new perspectives for learning science.

### Workshops

Biology-oriented programming workshops using Python for students and teachers were performed in one private and two public schools from La Plata, the capital city of the province of Buenos Aires, Argentina. These schools were selected on the basis of their willingness to take part in the project and the possibility to accommodate the instructors’ schedule in their weekly activities. Participation of the students in the workshops was voluntary in all cases, although framed in the context of a particular science course like biology or genetics. Although representing schools with different educational goals and infrastructures, the three groups of students that took part comprised similar demographic profiles, with mixed socioeconomic backgrounds and a balanced gender ratio. The workshops were delivered as three weekly 90-minute-long face-to-face classes offered during consecutive weeks, with one teacher or teaching assistant per ten students. They took place in the schools with the technological resources available at each of them, using installed versions of Python 2.7 and/or 3.6 on PCs and students’ smartphones. Online Python terminals (http://repl.it/languages/python3, http://www.tutorialspoint.com/execute_python_online.php) were also presented in order to show additional ways to use the language. Internet was only requested for the first meeting for Python installation. Overall, more than 90% of the students completed the practical exercises on their smartphones whereas the rest used netbooks or notebooks.

The workshops were aimed at students of the last years of their secondary school (a five/six-year-long stage equivalent to high school in the United States) due to the science background needed to face the biological problems presented during the course. In spite of the public or private nature of the school, the curriculum design in the province of Buenos Aires establishes a common core in natural sciences and mathematics. Apart from this shared nucleus, the students can follow different orientations with additional workload in distinctive subjects (a detailed description of the curriculum, in Spanish, is included in http://servicios.abc.gov.ar/lainstitucion/organismos/consejogeneral/disenioscurriculares/). In particular, the different activities proposed during the workshops require basic operations in mathematics, understanding of logical operators, and knowledge of the classical perspective on the molecular basis of information flow from DNA to proteins. Because these contents are included in the shared nucleus of all orientations, students from both years in each school joined a unique, integrated class for the workshop. The contents covered in each class are shown in Table 1.

**Table 1:** Course topics and organization.

Class	Contents
I	• **Hardware and software concepts.** What is programming, and what is it used for? • **What's new about Python?** Presentation of Python. Installation, online terminals, and mobile applications. Possible uses of the language. • **Python as a powerful calculator.** Basic mathematical and relational operators. • **Interaction with users.** raw_input() and others.
II	• **Quick note about errors.** Basic feedback and how to use it. • **Strings.** Manipulation and functions, e.g., replace(), upper(), lower(). • **Genes and proteins.** Biological sequences as a succession of characters. • **Arrays.** Manipulation and functions, e.g., append(), remove(), sort(). • **Membership operators (*in* and *not in*).** Searching for patterns in a big sequence.
III	• ***if* statements**. Write more complex programs by conditional execution of code. • ***for* loops**. Run code repeatedly for more complex programs. • **Making it better**. Editing existing programs in order to optimize them.

An interactive guide was given to students (see S1 File) in which many exercises were proposed. Some of them were taken as examples to solve during the workshop by the students with teachers’ assistance. Possible solutions were shared and evaluated collectively in order to take the maximum advantage of every different proposal.

After finishing the workshops, the participants were offered to take part in a bioinformatics challenge that was set up as a contest. Each school could present multiple groups of up to five students accompanied by a teacher. Three problems (see S2 File) were given to the students to be solved in a three-week period, during which the groups were monitored by teachers and workshop trainers. The exercises were written with increasing complexity, and each had extra goals to tackle in order to encourage a deeper analysis for working solutions. For example, the first question asked the participants to construct an algorithm for translating a hidden message between nucleotide and amino acid alphabets using the standard genetic code, with additional points awarded for showing the number and identity of codon sequences that could encode the message. Each group delivered their scripts and a written report detailing the general approach they applied, the difficulties they faced, and the major decisions they took toward their goal. Submissions were evaluated by an ad hoc committee. They provided feedback on early versions of the work and ranked the final submissions by testing that the programs worked as intended and evaluating the extra effort put into solving the optional exercises and the attention to documentation, presentation, and general style of code. All the examples of the final scripts built by the students answering the required questions are shown in S2 File.

### Smartphone applications

There are many smartphone applications (apps) available for the different operating systems (OSs), which may be more or less useful for working in the classroom, depending on the type of tools to create. When developing simple tools that could be run from the interactive interpreter or by loading single scripts, and that only require standard libraries, distributions that offer a Python terminal are sufficient and recommended. There are many Python apps available for Android and iOS, both free and paid, but fewer options can be used in Windows smartphones and are generally not optimal for running external scripts. In Table 2, we summarize some useful free apps for the classroom, among which QPython and QPython 3 for Android, Python 3 for Windows, and Python 2.5 for iOS were recommended to the students because these proved to be stable and responsive based in our preliminary evaluation in several smartphone platforms. These Python applications allowed students to test the code proposed in class quickly and easily, making the overall experience less passive and noticeably more engaging. Other more comprehensive apps may be needed in complex scenarios, especially if there is the need to load big external data files or use third-party libraries with multiple dependencies.

**Table 2:** Characteristics of suggested smartphone applications for running Python. Selected applications classified by OS and Python version. All programs are free and available in English.

OS	Application	Python version	Download link	Integrated terminal	Support external scripts	Help / Support
Android	QPython	2.7	https://play.google.com/store/apps/details?id=org.qpython.qpy	Yes	Yes	http://www.qpython.org/document.html http://edu.qpython.org/
	QPython3	3.6	https://play.google.com/store/apps/details?id=org.qpython.qpy3	Yes	Yes	
	Pydroid 3	3.6	https://play.google.com/store/apps/details?id=ru.iiec.pydroid3	Yes	Yes	No
Windows Phone	Python 3	3.6	https://www.microsoft.com/en-us/store/p/python-3/9nblggh083nz	Yes	Yes	No
iOS	Python 2.5 for iOS	2.5	https://itunes.apple.com/us/app/python-2-5-for-ios/id577916777?mt=8	No	Yes	In-app Python documentation
	pythoni	3.3	https://itunes.apple.com/us/app/pythoni-run-code-autocomplete/id493505744?mt=8	No	No	No

**Abbreviation:** OS, operating system.

### Python performance in smartphones

**Fig 1:**
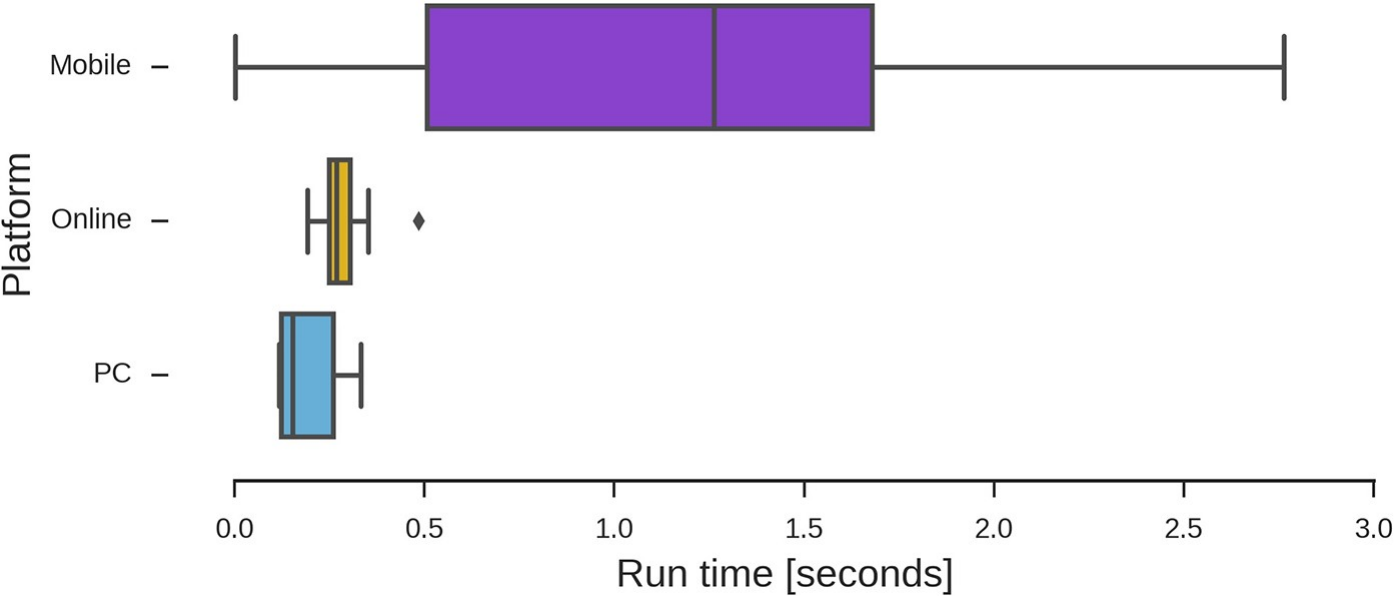
Distribution of calculation times on different computing platforms. The sample script used for testing (described in S3 File) is a modified version of one solution submitted by students to the contest. A total number of 25 different smartphones were used for the test and compared with desktop computers and Python on line distributions.

For a preliminary evaluation of different standard smartphone platforms, we tested several Python apps using a simple script and recording its calculation times. The script we implemented (see S3 File) is a “Translator” that receives a phrase in “human language” and translate it into “cells language.” Using the universal genetic code and the standard one-letter amino acid representation, most letters from the English alphabet could be written as one or more codons. Any word using these letters can therefore be translated to a large number of codon combinations. This idea was later explained and proposed to the students as an exercise too. The main purpose of this evaluation was to compare calculation times between PCs, online resources, and mobile smartphones that are available for students to perform bioinformatics calculations in a classroom. Our intention is not to benchmark smartphones performances, which depends on too many variables that could not be addressed here, but to assess whether those smartphones that are commonly accessible to students in our local communities would be able to complete the proposed tasks efficiently. From Fig 1, it is possible to infer that smartphones are on average slower than PCs for calculation times. Online resources such as Repl.it (https://repl.it/) can perform somewhere between PC and mobile phones, although permanent access to the internet should be provided.

**Fig 2:**
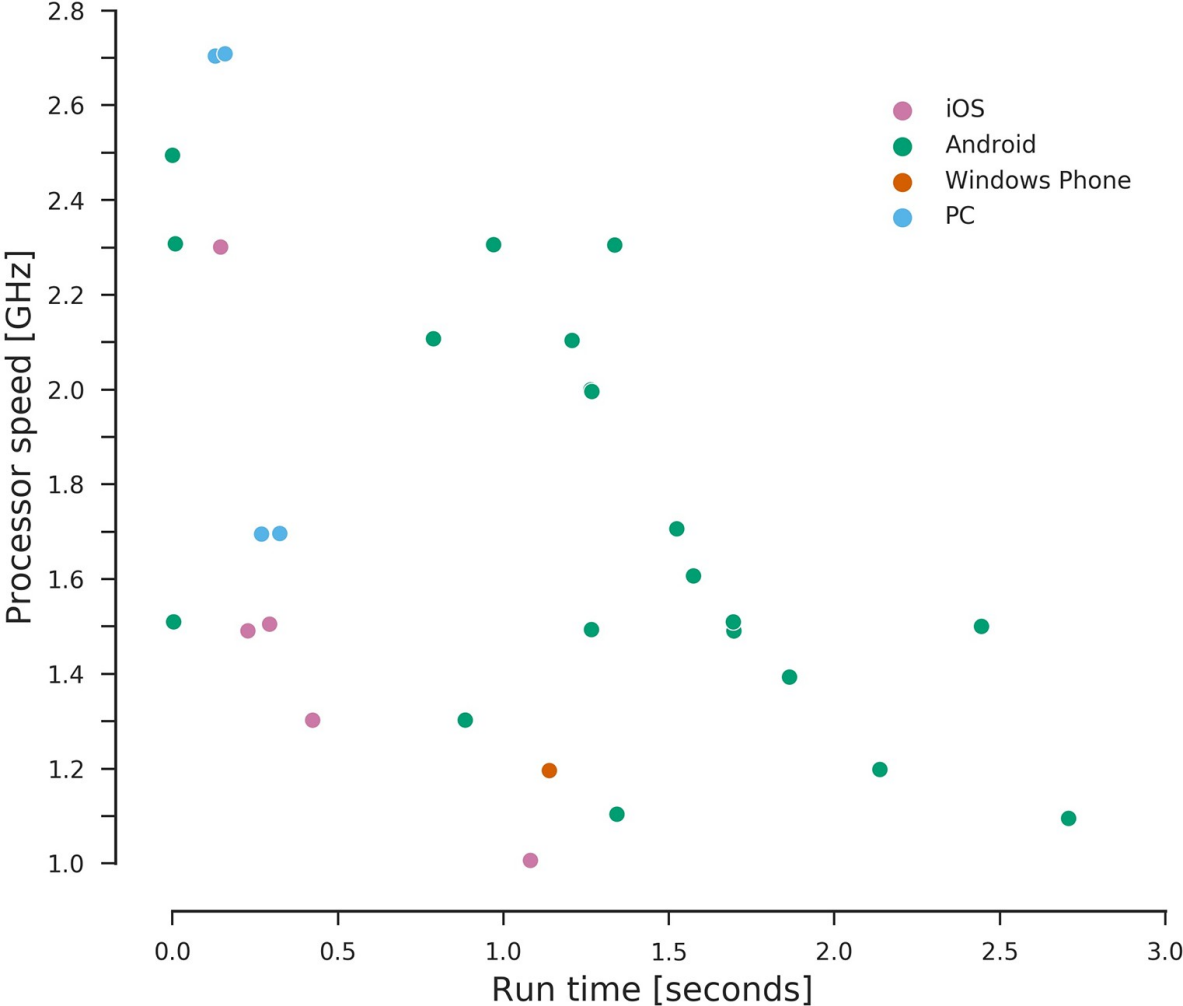
Distribution of calculation times on different processor speeds. Dots represent the mean over ≥5 replicates per experiment. Colors indicate the OS. OS, operating system.

**Fig 3:**
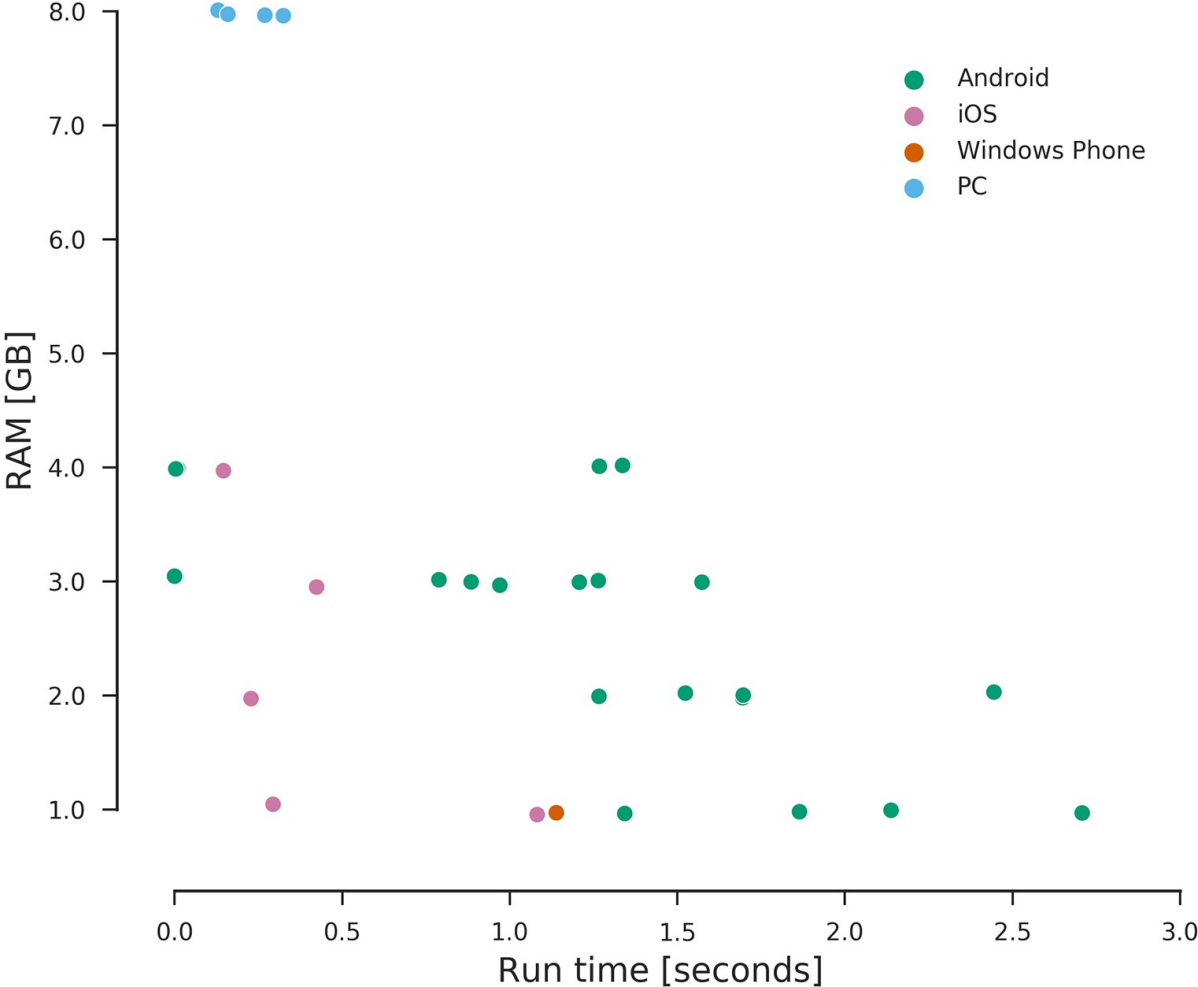
Distribution of calculation times on different amounts of RAM memory. Points represent the mean over ≥5 replicates per experiment. Colors indicate the OS. OS, operating system.

It is also possible to see in Fig 1 that some smartphones could perform even better than current personal computers. This does not seem to be highly dependent on the OS of the phone but on the available processor speed (Fig 2) and RAM memory (Fig 3), with other factors such as the Python interface used and the system load possibly affecting the running times. According to our results, smartphones were at most an order slower than a typical desktop PC hardware setup (Intel i5-6400 2.7GHz quad-core processor with 8Gb RAM), proving very capable of serving as programming tools for this kind of course.

## Discussion

A total of 100 students aged between 16 and 19 years old were part of the project, all of them owning a smartphone; 92.9% of the students didn’t have previous programming knowledge, and most of them (87.5%) did not know about bioinformatics. More than half (53.57%) of the participant students were from the natural sciences orientation. Students enrolled in the social sciences (35.71%) and economy (10.72%) orientations also took part in the workshop, showing that the proposal of learning a programming language was transversal and attractive for the students in general.

The tools chosen for the workshops (smartphones apps and online terminals of Python) made the teaching–learning process, as well as the exchange of knowledge among students, engaging and effective. As derived from student’s feedback, the exchange of ideas was fluid and the immediacy provided by these technological devices allowed students and teachers to explore different variants for the proposed exercises, generally derived from questions raised in the classroom, making it possible to evaluate several possible paths toward a solution. The biological questions proposed and solved in the workshops (see Table 3) triggered challenges in programming and enriched the overall learning–teaching process.

**Table 3:** Some biological questions made during workshops and the proposed programming task to address them.

Biological question	Programming task	Section in supporting information
What are genes and proteins?	Randomly insert mutations of a certain type, on a given string that represents a DNA sequence.	Workshop challenge 5
What is a mutation?		
What could be the effects of mutations on genes and proteins?		
What is the relationship between similar genes and proteins in different organisms?	Compare the lengths of two strings that represent related gene sequences with mutations	Workshop challenge 7
What makes genes and proteins different from each other?	Find given functional motifs within one or more gene sequences	Workshop challenges 8, 9
What are the defining features that could be found in genes and proteins?		

Our results show that the use of smartphones could help to surmount the limitations related with the availability of computers in high schools. The easy setup of this kind of workshop, based almost entirely in smartphones and thus independent of the available equipment in schools, triggered a great interest of the educational community and generated enthusiastic responses in students. Although it is yet not possible to collect enough evidence to address the impact of our workshops, this novel approach should let students deepen their knowledge and interest in the field by revisiting biological concepts under a new light. The workshop should have also helped students to realize the potential of acquiring programming skills, giving them a tool not only for understanding and experiencing science, but also for developing strategies to help solve different challenges of their future professional life. Altogether, we think that these practices reinforce the notion that bioinformatics provides a suitable framework to improve the learning-teaching experience of biology and programming.

## Supporting information

S1 File Theoretical and practical workshop guides. Guide given to the students for learning Python programming oriented to biology.
(DOCX)

S2 File Contest questionnaire and students’ solutions. Contest questionnaire and examples of final scripts built by the students answering the required contest questions.
(DOCX)

S3 File The “Translator” script. This script was used for testing different platforms—multiple combinations of smartphones models or PCs, with different OSs and Python versions, or online tools. OS, operating system.
(PDF)
