# Supplementary material for: Bioinformatics calls the school: Use of smartphones to introduce Python for bioinformatics in high schools
Source: PLoS Comput Biol. 2019 Feb 14;15(2):e1006473. doi: 10.1371/journal.pcbi.1006473 (PMC6375546; doi:10.1371/journal.pcbi.1006473)
Supplement: S1 File — Guide given to the students for learning Python programming oriented to biology. (DOCX) [file pcbi.1006473.s001.docx]

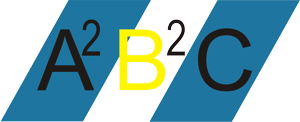

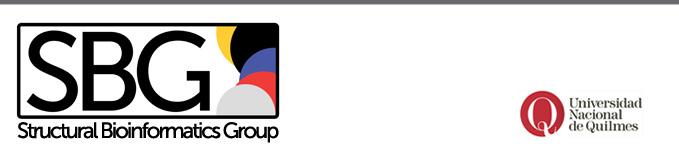

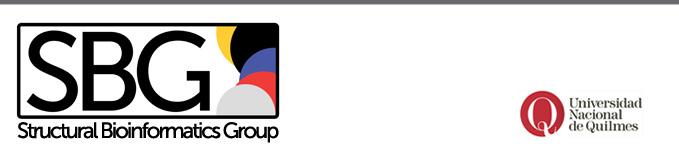


**WORKSHOP: BIOLOGY-ORIENTED PROGRAMMING**

**(Adapted from the original version in Spanish)**

**CONTEXT**

Bioinformatics is a scientific area that uses computational methods for the analysis of biological data in order to answer biological questions. The use of computational techniques allows, for example, the analysis of large datasets from experimental observations, reference publications or public databases, in short terms of time; the simulation of complex behaviours of biological systems; or the prediction of the activity and function of different biomolecules. Besides being a field of study within biological sciences, Bioinformatics may be thought of as a tool for teaching and learning biology, since its methods allow the ‘visualization’ of different processes inherent to biological entities (DNA, proteins, cells, whole organisms and their populations, etc.). Thus, Bioinformatics generates a significant and comprehensive learning experience by providing, at the same time, the tools needed to integrate multiple aspects of knowledge (logical-mathematical, biological, physical, statistical) and an alternative way of perceiving biological processes.

**Welcome! Are you ready?**

Before we begin, we need to agree on some definitions:

**What is a computer?**

The physical elements of a computer are called hardware, while the software corresponds to the instructions needed for the correct operation of the hardware. The operating system provides an interface between the software and the user and allows the rest of the programs to interact correctly with the hardware.

**What do we do when we program?**

A programming language is an intermediate one between the language of the user and that of the computer. If we could describe a computer in a very simple way, we could say it consists of a large number of electrical circuits that can be activated or deactivated. We say computers use a binary language: their only ‘letters’ are ‘1’ (indicating ‘on’) and ‘0’ ( ‘off’). The user interacts with the computer by setting different on-off combinations of these circuits that the computer can understand and apply to perform some action, like displaying something on the screen. Instead of learning the native machine language, a programming language can be used to give instructions to the computer in a way that is easier for humans to learn and understand. Then an intermediate program, called compiler, translates instructions from the programming language into machine language**.** Thus, as a programmer, it isn’t necessary to understand what the computer does, or how it is done, but just to know how to ‘speak’ the programming language.

**Why learn to program is useful?**

Computers, smartphones and gaming consoles wouldn’t be very useful without programs to make them work. Each time we open a file in the computer, send a message on WhatsApp or watch a movie on the phone, we are using programs that interpret what we want (“send message to Eva”) and make the connection within our computer or smartphone to execute the order. Programming allows you to do an enormous amount of different things: from writing your own games or reading thousands of texts without opening a book, to analyzing the complete genome of an organism or estimating the evolutionary history of millions of proteins.

**What is Python then?**
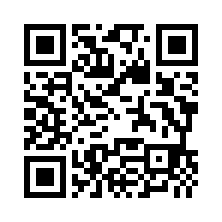


Python is a programming language with a syntax that is easy to read and write for humans. In particular, Python is a scripting language, which can be executed in blocks and without the need to run a compiler. The most important reason to choose Python now, is that you can learn programming and jump straight to writing your own scripts really fast. However, Python has many features, advantages and uses that won’t be shown in this short workshop, but we encourage you to discover about them from the official Python and Python Argentina websites.

**How do we use Python?**

It depends on the device we have. On a computer, it depends on your operating system:

**Windows**
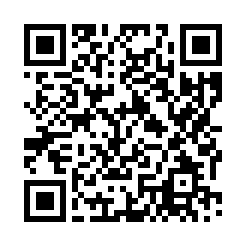


You can download Python for Windows from the official Python website (<https://www.python.org/downloads/> ). After downloading the *.msi file, run it and follow the instructions to install.

It is important that you remember the path to the folder where Python has been installed. We recommend you to click on the option “Add python.exe to the path” in the second screen (“Customize”) of the wizard.

**Linux**

It is advisable to verify if you already have Python installed, which is very likely in modern Linux distributions, and what version it is. You can do this by opening the Unix terminal and writing:

$ python2 --version

and

$ python3 --version

Although Python 2.X and Python 3.X are currently used, and most of the basic code would work in both versions, there are significant differences between them. Any of these versions can be used in this workshop.

If you have Ubuntu and you don’t have Python installed, or you want a different version, you can get it by writing in the terminal the following command (modify python3.4 to your preference):

$ sudo apt-get install python3.4

**On your smartphone**


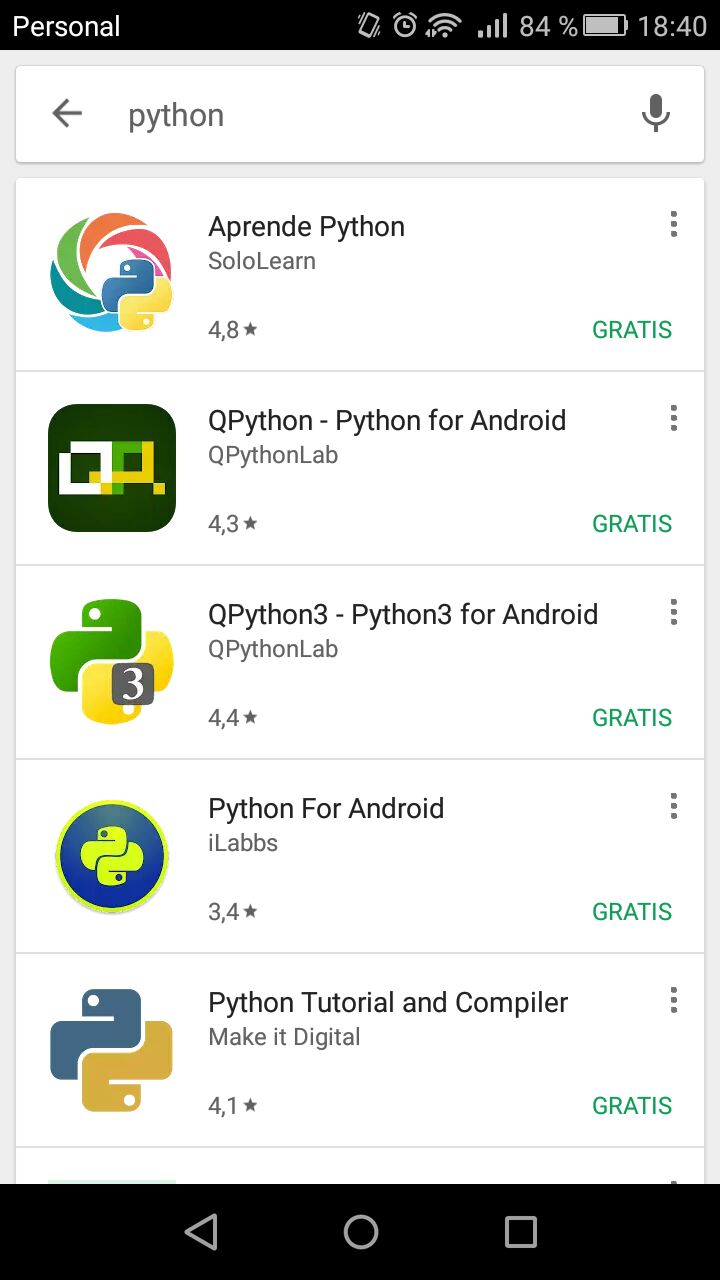


Bad news! When using a smartphone for programming, you have to find the correct distributions too, which also depends on the operating system of the phone. Fortunately, free apps exist for the different systems! You just need to search “Python” in the store and download the right one.

- For Android: ‘QPython’ (the Python 2 version) or ‘QPython3’
- For Windows 10 Mobile: ‘Python 3’
- For iOS: ‘Python 2.5 for iOS’ or ‘Pythoni’

**What other ways are available to use Python?**
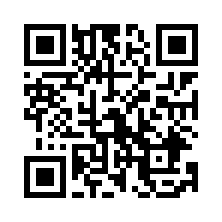

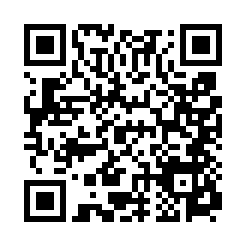


There are online terminals which allow you to use Python in a similar way as if you have it installed on your computer. They are totally free, though they require Internet. We recommend the following two options but you can look for others if you want:
repl.it: <https://repl.it/languages/python3>

tutorialpoint.com: <https://www.tutorialspoint.com/execute_python_online.php>

**Jupyter Notebook and Anaconda: a friendly interface for running Python on your computer**
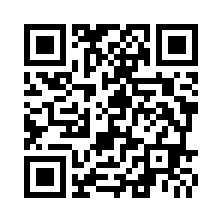


Jupyter is what is commonly called a Python commands interpreter. It is an interactive and friendly environment that facilitates the programming process: it does not just offer a black terminal, but it has buttons to navigate files on our computer, run programs, undo actions, etc. Jupyter can be used for free and there are distributions for (almost) all operating systems (<https://www.continuum.io/downloads>). Jupyter has built-in libraries that are very useful for scientific data manipulation. It is also friendly for data navigation and code testing, since blocks of content are distributed in cells that can be executed individually.
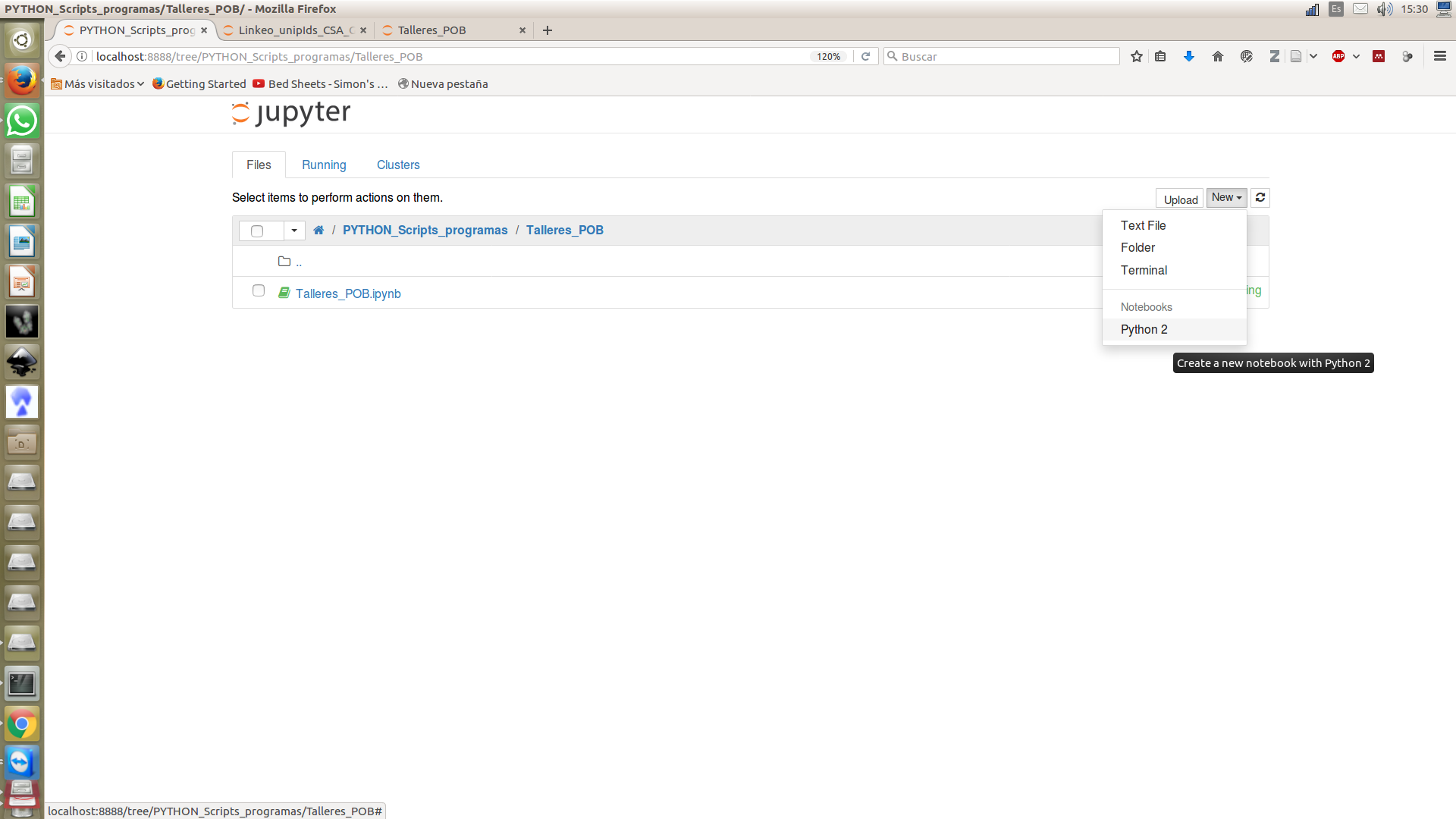


If you want to use Jupyter on Windows systems, remember that the installation must be done for all users and keep in mind that the path where it is installed cannot have spaces (for example: C:\Users\Me\Documents\Anaconda2). To run it, click the Windows button and search for 'Jupyter Notebook', then execute it by clicking in its icon. A terminal will pop up and a new tab should open in the Internet browser (we recommend Chrome). This tab is the Jupyter interface.

This interface allows you to explore the files in your computer (“Files” button), create new text files (New → Text File) or generate '.ipynb' files, known as Notebook files (New → Python 2), where all the information of the session in which you have been working in is stored. The Jupyter environment allows you to use these files interactively, creating cells where you can execute the code that we want to test (“+” button), eliminating cells that you don’t want to keep in the file (“cut” button, scissors), or saving everything you have written (“save” button, the floppy disk), etc. To execute each cell with the code that you wrote inside, just do SHIFT + ENTER. Here we don’t see the Python prompt (explained below), but we will know when the cell is executed because we will see on the left side "In [*]" and when the execution finishes, "In [1]" (Where the number within the brackets is the execution number, so when you run a cell after it, "In [2]" will appear.)
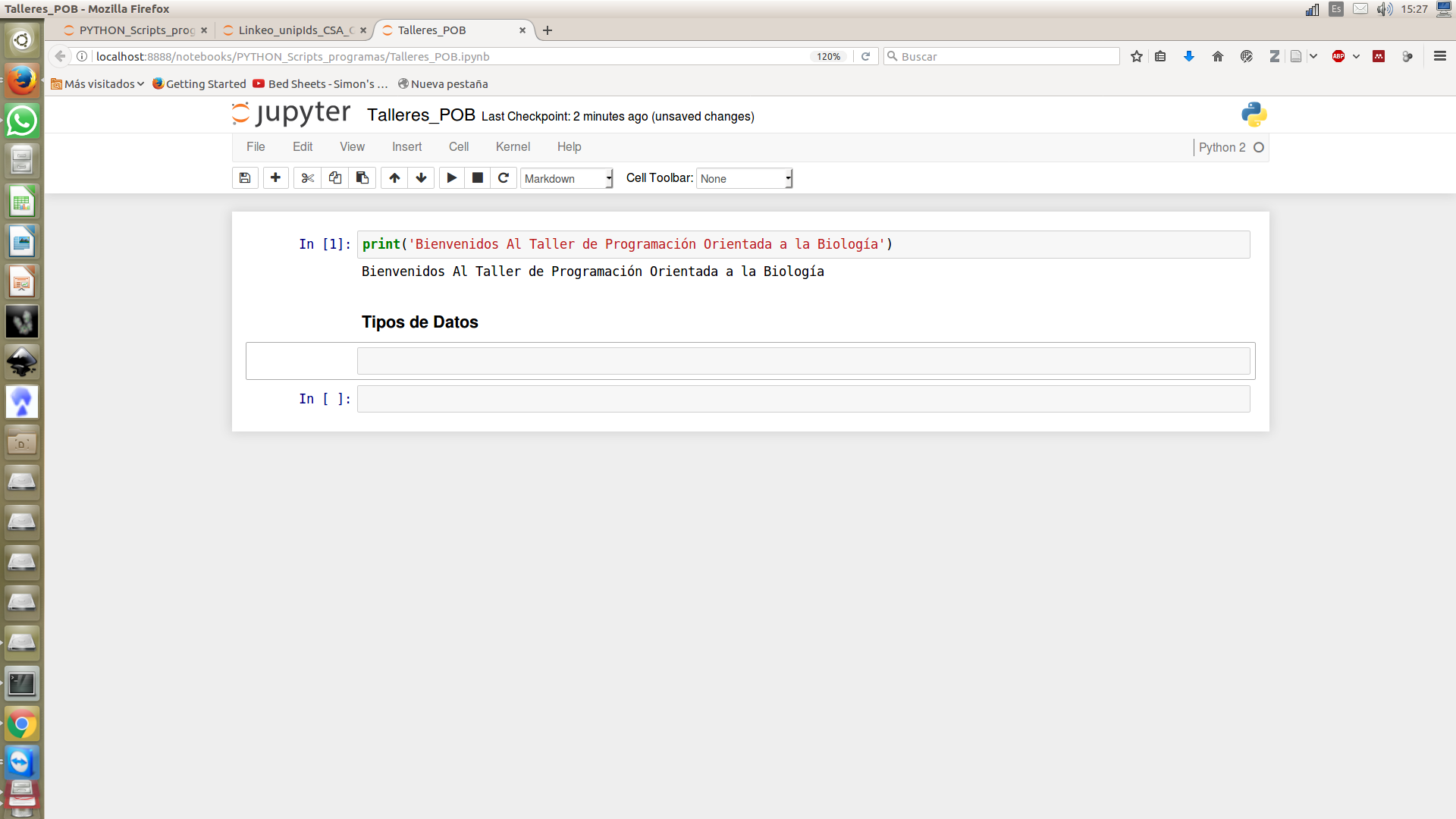


**The journey of a thousand miles begins with a single step...**

A first step for making your first program is opening the Python console on your computer, Python app on your smartphone or online console:

If you are using a **UNIX** computer, open a Linux terminal (by doing Ctrl + Alt + t in Ubuntu) and write:

$ python

If you are working with the Python interpreter Jupyter you must write:

$jupyter notebook

If you are using **Windows**, the console opens from the Windows start button. Search the Python 2.7 folder (or any version you have installed) and execute Python Prompt by clicking on its icon. If you are working with the Python interpreter Jupyter you must look for ‘Jupyter Notebook’ and execute it by clicking its icon.

If everything went well, you will see the Python "prompt", in which there are three major signs ">>>". The prompt always appears when Python is ready to receive a new order.

If you are using a smartphone, open the console in your Python app.

**The start of a beginning…**

In the learning process ‘mistakes’ have a very important role: they generate new questions, take us closer to new hypotheses and, most important of all, they give us new opportunities for keep on learning. In programming, ‘mistakes’ or ‘errors’ are not only important, they are very important! They are a kind of communication with the machine that warn us when something we are trying to do isn’t working. There are different types of errors in Python and each of them reveals what could be going wrong with our code. These are the reasons why we ask you to take note of every error that might appear throughout your work during the workshop (in-person or at home) and we encourage you to share them with us, so we can take the opportunity and discuss them with the class, in order to enrich the solutions we come to. **So, as Ms. Frizzle from The Magic Schoolbus would say: Take chances, make mistakes, get messy and rock it with Python, where the road ends, adventure begins!**

**Your first program**

A not so original way to start programming is simply writing on the console the next sentence followed by enter:

print(‘Rock it with Python!’)

So, what happened? Yes, print is a function that allows you to show on the console everything which is written between quotes (single or double quotes) and within the parenthesis (as in our example). This function, among other things, allows us to interact with our program or the future user of it. Congratulations, that was your first program in Python!

**A super cool calculator**

With Python we can do all sorts of math calculations! Even though it may sounds quite boring, many of this calculations will help us later with a lot of different kinds of data. Let’s start then, write on your console:

3*5

What is the result? As you can see, the asterisk is the symbol used for multiplying in Python language. Let’s try now:

8/4

What result did you obtained? What’s the forward slash used for?

Basic math symbols (usually called operators) that can be used in Python are:

| + | Addition |
| --- | --- |
| - | Subtraction |
| * | Multiplication |
| / | Division |

What outcome would we obtain by doing the following operation?

5+30*20

What happen if we now write the operation as follows?

(5+30)*20

What’s the result? Why do you think it’s different from the previous one?

So, from both previous operations we can conclude two important things: in this language, as in maths, operators (math symbols) don’t have the same priority regarding reading or executing. Multiplication and division have a higher priority than addition and subtraction. This means that in the first example 5+30*20 Python executes 30*20 first and then adds 5. The parenthesis helps then to reorder Python priorities. By writing (5+30) we force the execution of this operation to be the first.

Let’s try something more complex?

((4+5)*2)/5

**CHALLENGE 1: Can you find out and write down the order in which each operation was executed?**

**Slow down egghead!**

A variable is a space reserved in your computer’s random access memory (RAM), to store modifiable data.

So, when we declare and assign a value to a variable, we are saving it in the memory. We are grouping ‘things’ and relating them with a name (variable), then once the variable is declared Python remembers that the assigned things are inside that variable. This term is used in every programming language. In Python, a variable is defined by the sentence: variable name = variable value.

Let’s define some variables:

a = 5

b = 3

c = ‘hello’

As you can see a variable can be a number, a text, a list of things, etc. In the case of words or text it always has to be written between quotes (double or single) so Python considers it as a text. If we now ask Python what b is?

print(b)

We’ll see that it has been properly saved in the memory that b is 3, as Python shows in screen the value of 3 when printing b. In this programming language, we can overwrite variables and assign a new content by simply declaring them again:

b = 500

print(b)

We can also assign the same value to more than one variable

d = 500

print(b)

print(d)

**CHALLENGE 2: Create a variable called double, which is double of the sum between a and b.**

**What about variables?**

This issue about ‘variables’ is very interesting but, what is it useful for? An obvious usage is to store data in the memory, which can easily be forgotten and needed in the future. A variable could also help us to store data we don’t know in advanced but that we’ll want to modify or use once it is acquired. Let's suppose that we would like to create a program that greets the user. We don’t know beforehand who is going to use it, but we could ask their name first and then greet him or her, right? To do that it could be useful to save the person’s name:

raw_input(‘Tell me your name please!’)

This function raw_input asks the user for some data (by printing in a message, whatever we write between quotes within the parenthesis) and waits for an answer. You will see that once the line is executed, the *prompt* doesn’t come up again until we write something and hit enter.

Now, could you make a program that greets the user?

Let’s think together the steps necessary to do that:

1. The first thing we should do is to ask the user his/her name and store it in a variable
2. Then we could print a message on the screen that says ‘Hello ….., welcome to my program!’

Let’s try it:

user_name = raw_input(‘Tell me your name please!’)

print (‘Hello’ + user_name + ‘Welcome to my program!’)

It is very likely that if you did this on your own, your program isn’t exactly like this one above. When programming, there are many ways to get the same result! So, if your program greets its user you did a good job, even if it is not the most stylish syntax program. As you can see, the program we wrote uses a trick to print the greeting, which could be useful for you: in Python we can join words or text to make longer sentences by simply adding them (+), as we did in the example.

**Why is a raven like a writing desk?**

Now we know how to declared variables and assign them values, there are also different ways to compare them and perhaps understand if a raven is like a writing desk! The symbols used for comparing variables are known as *relational operators*. We can know if two variables are the same (==), or if one is larger than the other (>) or if they’re different (!=). For example:

one_thing = ‘writing desk’

second_thing = ‘raven’

print(one_thing == second_thing)

What result do you obtain when comparing two variables?

Yes, *relational operators* do not give numeric values as a result, they either affirm (True) or reject (False) whatever hypothesis we were testing. In our example, the hypothesis is an equality between one_thing and second_thing (in this case words), and what Python will give us as a result is a rejection: False.

Relational operators that can be used in Python are:

| == | Equal |
| --- | --- |
| != | Different |
| < | Lesser than |
| > | Greater than |

Numbers can also be compared:

print(5 > 3)

**More than words…**

In programming, text data are called ‘strings’. This type of data is nothing more than a chain of characters, just like a word is nothing more than a chain of letters, thus a string doesn’t necessarily have to make sense. In Python strings are defined by writing characters between single or double quotes and can be assigned to a variable as follows:

chain = ‘this is a chain example’

print(chain)

We can print a string with another string embedded by using the marker **%s**, which marks the place where the text is going to be incorporated.

For example:

my_text = ‘Hello %s’

print(my_text %s ‘Ana’)

Something similar can be done with numbers:

the_result = ‘The result of the calculation is %s’

print(the_result %5)

We can add and multiply strings in the same way than operating with numbers.

a = ‘Hello’

b = ‘Kids’

print(a+b)

What if we add a number to a string? Try writing print (a + 5) on your terminal and see what happens!

**CHALLENGE 3: How would you write a program that says ‘*I love you’* 100 times to someone?**

Strings can be compared as we did before using relational operators, so we can know if two strings are different or not (keep in mind that Python differentiates uppercase and lowercase)

word = ‘yes’

the_same = ‘yes’

print(word==the_same)

What if we write the variable ‘the_same’ with uppercase?

**Slices of text!**

In Python we can access or know which are the characters or sub-parts of a string. Python assigns a position number to each character within a string. First character of a string is position zero (yes, zero!) and so on, until the chain ends. For example in the following string: a=’Hello’, ‘H’ has the position zero, ‘e’ the position one, the first ‘l’ the position two, the second ‘l’ the position three and ‘o’ the position four. So if we want to know which one is the first character of this string, we can refer to the position zero by writing the name of the variable followed by the position number we’re interested in, enclosed in brackets:

a = ‘Hello’

print(a[0])

We could also take just one segment of the chain indicating, between brackets, the start and end positions separated with a colon:

a[0:1]

**Chamuyo: From the Lunfardo, the art of manipulating words/strings.**

There are many useful functions to manipulate strings. As we said before, Python is case sensitive. We can use some functions to interconvert between upper and lower case.

surname = ‘velez’

surname.upper()

what if we want lowercase?

name = ‘ANA’

name.lower()

We can also obtain the length of a chain (how many characters it has) by using the **len()** function:

surname = ‘velez’

len(surname)

**CHALLENGE 4: Can you create a program that asks the user for his/her name, greets him/her and says how long his/her name is?**

Another useful function to manipulate strings is ***replace()***. This function allows us to replace one character with another. For example:

surname = ‘velez’

surname.replace(‘e’,’a’)

As you can see, for replacing a character with another you have to write between quotes and separated by a coma the character you want to replace and its substitute, everything enclosed in parentheses, as arguments for the **replace** function.

**What is essential is invisible to the eye...**

For Python, looking for a needle in a haystack is a childsplay! Performing the metaphor 'to read between the lines' is something that can be done without any trouble. Even for strings with one million characters, we could find character patterns in a few seconds and know if a substring (a fragment of a chain) is present or not. Membership operators ***in*** and ***not in*** allows us to perform this kind of search. Simply by asking if a certain substring is in the chain, Python responds True if it is present or False if not:

chain = ‘Okilly-dokilly!’

‘oki’ in chain

**Nature’s language**

When dealing with biological data, strings are very frequent. Much of the data that we can obtain about an organism or about a biological system, can be thought as a sequence of characters or 'words': DNA, RNA and protein sequences, etc.

**CHALLENGE 5: Let’s assume that we have the sequence of a gene (which is nothing more than a sequence of characters that, in the case of DNA molecules, are called nucleotides) of a mini-protein (amino acid string) from a frog with hair, brought from Mars. And we know that a song by Ricardo Arjona may generate mutations in its DNA. Each time ‘Pingüinos en la cama’ sounds, a Thymine (T) changes to an Adenine (A). Knowing that the gene sequence originally was ‘ATGGAAGTTGGAATGGTTGGAATGGTTTTAATTGGAATGGTTTTAAAAGGTGAGTGGTATTTGATGATAGTTGGATTGGAATGGAAGTTG’, can you design a program that tells you how is the gene going to be like by the end of a music playlist, where the song is repeated twice?**

**Gee, Brain, what do you want to do tonight?**
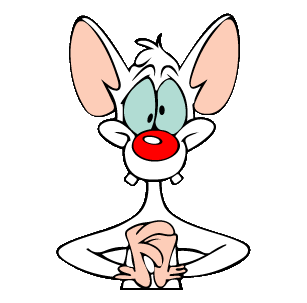


Obviously, as Pinky and the Brain usually say, our biggest goal in life is to conquer the world and for that we have to think big! For this reason it’s always useful to know how to handle a big amount of data.

Python allows us to work with a type of data called lists, which are groups of data (chains or numbers). So, for example, if we want to keep track of how many countries we have conquered, and of course they are a lot, we could save their names in a list, so we don't have to remember them:

conquered = [‘Argentina’, ‘Uruguay’, ‘Brazil?’]

Lists are written by placing the elements within brackets and separated by commas. The elements can be strings or numbers:

list = [1,2,4,100]

We can access the elements of a list in the same way we access to string elements, by indicating with a number between brackets the position of the element we want. Remember that Python starts counting by zero:

list[1]

We can select some of the elements on the list by doing:

list[0,2]

And also, as we did with strings, get the number of elements on the list (its length) with the **len()** function:

len(list)

**Step by step, bit by bit...**

A resource commonly used in the development of programs is to create empty variables that can be filled later. Let's imagine that Pinky and the Brain share with us their evil plan to conquer the world. Assuming we start tonight, our list of conquered countries would be empty, and while we are becoming the masters of the world, the list will expand! So how can we make an empty list? We simply declare a variable with a list format but empty (nothing in it):

conquered = []

Once the variable is declared, we can add the conquered countries by using the ***append()*** function:

conquered = []

conquered.append(‘Argentina’)

conquered.append(‘Uruguay’)

conquered.append(‘Brazil’)

If one of the conquered countries is free again and we lose allies, we could remove elements from our list by doing:

conquered.remove(‘Argentina’)

And, if we were very organized conquerors and want to keep a neat register, we can order the list of countries using the ***sort()*** function.

conquered.sort()

**CHALLENGE 6: Which operators could you use on lists?**

**The never-ending story...Almost!**

In Python language, there are types of data called Tuples and Dictionaries, both very useful when you are programming. But this time we are going to leave the investigation to you!

**Should I Stay or Should I Go...**

As in life, when it comes to programming, you must make decisions and these will always depend on the conditions that arise. In Python there is a sentence called ***if*** that allows the program to do one thing or another depending on the condition we have set beforehand. If certain condition is fulfilled, *then* the program does something and if it is not, we can ask it to do something else using the ***else*** sentence. The structure of the ***if*** statements would be something like this:

if condition:

here you write the order that is going to be executed if the condition is fulfilled

else:

here you write the order that is going to be executed if the condition is not fulfilled

As you can see, the sentence ***if*** is formed by an ‘*if*’ followed by a ‘*condition*’ followed by a colon (:). The next line (or block of lines), which has the order to be executed, always begins with a *tab*.

The **else** sentence is formed by an ‘*else*’ followed by a colon (:) and the next line also begins with a tab.

A condition is a programming calculation/analysis which results in ‘Yes’ (True) or ‘No’ (False) and it can be created, for example, by using the relational operators you already know (== , >, ≥,<, ≤, !=).

Let’s see an example to understand if/else sentences a little more: let’s assume we want to improve the ‘greeter program’ that we have made before. We can make it more interactive by making the program decide if the user’s name is long or short, depending on how many letters does it have. Let’s write the steps that our program should follow:

1. Ask the user its name and storage it in a variable.
2. If the name is shorter than 4 letters, the program will be surprised and tell the user that he or she has a short name.
3. In the opposite situation, the program will just greet the user.

name = raw_input(‘Hello! What's your name?’)

if len(name) < 4:

print(‘What a short name!’)

else:

print(‘Hello’ + name)

We could make our program even more complex: now it will be surprised if the name is too short (less than 4 letters) o too long (more than 7 letters).

name = raw_input(‘Hello! What's your name?’)

if len(name) < 4:

print(‘What a short name!’)

if len(name) > 7:

print(‘Wow, what a name!’)

**CHALLENGE 7: Now that we found the frog specimen with hair in Mars, we would like to contrast its features with earth frogs. Knowing that the tiny protein gene is ‘ATGGAAGTTGGAATCCAAGTTGGA’ and the gene of a similar protein from earth frogs is ‘ATGGAAGTTAATGGAAGTTGGAGGAGA’, can you implement a program that compares the length of both genes and prints a result saying which gene is longer?**

**CHALLENGE 8: In the last seconds invisibility researchers have had a huge improvement. There are untrustworthy results that point out to the presence of a nucleotide sequence ‘ACATAAA’ that could be related to invisibility in humans. Make your contribution to science by creating a program that detects this invisibility marker in a given gene, for example:**

**‘ACAATTTAGAAAGTTAATGGAAGGACATAAAAGTTAATGGAAGTTGGAGGAG**

**GGTTATCA’.**

- **CLUE: You could start using again the membership operators we saw earlier.**

**CHALLENGE 9: Can you improve your program by making it useful to analyze more sequences or to analyze a list of deoxynucleotide sequences?**

**seq 1 : ‘ACAATTTAGAAAGTTAATGGAAGGACATAAAAGTTAATGGAAGTTGGAGGAG**

**GGTTATCA’**

**seq 2 : ‘ACAATTAATGGAAGACATAAACATAATTGGAGGAGAGGAGACATAAAAAAACATA’**

**seq 3 : ‘ACAATTTAGAAAGTTAATGGAAGGACATAAAAGTTAATGGAAGTTGGAGGAG**

**GACATAATTTAAAAATTATCA’**

**seq 4 : ‘ACCGAGCCCCGAAAATAAAGTTAATGAAGTTAATGGACATAAAGTTGGAGGAG**

**GGTTATCA’**

**seq 5 : ‘AAAGGACATAACCAATTAAACATAGAAAGTTAATGGAAGTTGGAGGAG**

**GGTTATCA’**

**seq 6:**

**‘ACAATTTAGAAAGTATTTAGAAAGTACATAAATTTAGAAAGTTAATGGAAGGACGAGGAG**

**GGTTATCA’**

**It’s a loop!!**


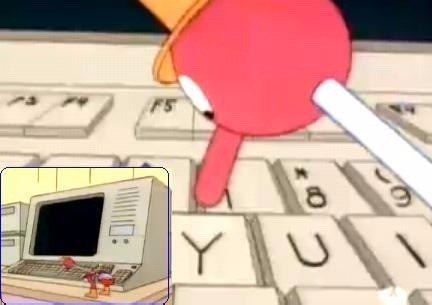
Some people are reiterative, some circumstances are reinteratives and some tasks are also reiterated. So unless we have Homer’s typing bird, knowing how to program in Python could make our lives easier! Python, as well as other programming languages, has what is called a for loop, that can save us a trouble. If, for example, we had wanted to express our love to someone by making a program for showing the message ‘I love you’ several times, we could had taken the long road:

print(‘I love you!’)

print(‘I love you!’)

print(‘I love you!’)

print(‘I love you!’)

print(‘I love you!’)

print(‘I love you!’)

And so on… which is kind of cumbersome… or we could had created a loop that says ‘I love you’ multiple times, in just two lines of coding, without the need of being writing the same thing over and over again:

for i in range(0,100):

print(‘I love you’)

The ‘lover program’ we have just made has a ***for*** loop, which consists of a ‘***for***’, a changing variable (in our case ‘*i’*), an ‘*in*’ and a list of things or a range on numbers that are the values that our variable ‘i’ is going to take. Our loop can then be read as:

For the variable i taking values between 0 and 100, print on screen the message ‘I love you’. But, how many times did our program printed the message? The ***range()*** function allows us to make, in a fast and easy way, a list of natural numbers that starts with the first and ends before the second number in the pair that we gave as parameters for ***range()*** (in our case, ‘i’ will finish by adopting 99). Remember, it is really important to leave a *tab* at the beginning of each line in the block of orders following the ***for*** sentence, on the contrary Python will let you know that you made a mistake, by showing you the error message:

**IndentationError:​ ​expected​ ​an​ ​indented​ ​block**

Indentation with tabs is the way in which Python recognizes the order or the orders to be executed as part of a loop or, as we also saw before, an if or else condition.

**Winter is coming…**

***For*** loops can also be written using lists. In this case, the values our variable ***i*** is going to take will be the elements on our list. Let’s imagine we are Arya Stark and while we dominate the magic of The Faceless Men we review our vengeance list: Weese, Dunsen, Polliver, Raff the Sweet, Tickles, The Hound, Ser Gregor, Ser Amory, Ser Ilyn, Ser Meryn, Joffrey, Cersei...We could make Arya’s vengeance easier if we built a program (‘Arya Stark’s conscience) that doesn’t let her forget who to hate:

hated = [‘Weese’, ‘Dunsen’, ‘Polliver’, ‘Raff the Sweet’, ‘Tickles’, ‘The Hound’, ‘Ser Gregor’, ‘Ser Amory’, ‘Ser Ilyn’, ‘Ser Meryn’, ‘Joffrey’, ‘Cersei’]

for i in hated:

print(‘You must take vengeance on’ + i)

**Spoiler Alert!**

Let’s assume some of Arya’s enemies are already off the list for higher reasons, such as Joffrey, and we want to make a different comment (‘One less to go!’) when the program ‘Arya Stark’s conscience’ reaches that element on the list. For that, we should:

1. Define hated list.
2. We want to check the elements on the list in a loop but:
3. If the element is Joffrey we must print on screen ‘One less to go!’.
4. In the opposite situation we must print ‘You must take vengeance on’ whoever it is.

Apparently, a simple *for* loop is not enough to reflect Arya Stark’s complex thoughts. So we should ‘nest’ the *for* loop for a condition ***if*** so it distinguishes between one element on the list and another, and our program can take decisions:

hated = [‘Weese’, ‘Dunsen’, ‘Polliver’, ‘Raff the Sweet’, ‘Tickles’, ‘The Hound’, ‘Ser Gregor’, ‘Ser Amory’, ‘Ser Ilyn’, ‘Ser Meryn’, ‘Joffrey’, ‘Cersei’]

for i in hated:

if i == ‘Joffrey’:

print(i + ‘One less to go!’)

else:

print(‘You must take vengeance on’ + i)

Let’s review a little what our program is saying:

For each person in the hated list (for i in hated), if the person is Joffrey (if i == ‘Joffrey’) we celebrate (print(i + ‘One less to go’)), if it’s not Joffrey we remember to take vengeance (print(‘You must take vengeance on’ + i)). Pay attention to the spaces after the *for* loop and *if* sentences colon (:), remember these spaces are important so Python can follow the orders we give in the correct order. If we don’t respect these tabs Python will let us know is not happy!

**CHALLENGE 9: Do you think you could write an interactive game that asks the user for some information? Is time to create: Take chances, make mistakes, get messy and rock it with Python, where the road ends, adventure begins!**

**What else can we do with Python?**


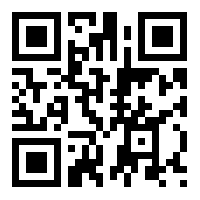
As we told you in our workshop presentation class, Python is a very versatile language which allows us to make all sorts of things: from simulations and complex calculation programs to web apps, android apps, data mining, etc. Everything about Python is open access, including libraries and tutorials and you can always ask us any doubts you have.

Remember, the trick for being a good programmer is to know how to google! You can look for every mistake you come across when you execute your programs on the web or in pages likes Stack Overflow (a useful website for programmers that work with all kind of programming languages, where you can ask questions and find information) (<https://stackoverflow.com/>) .

**This is not all folks!**

This is how far our workshop will go! We would like to thank you for your interest and participation. We’d like to thank the schools that opened their doors to us and gave us the opportunity to bring a little of our work closer to you.

We also want to thank the teachers that made a space in their curricula and let this project be carried out. We invite you to join our group and visit our website whenever you wish. **We’ll wait for your participation on the I Bioinformatics for High Schools Contest!**

​**Total Thanks...**

**The SBG team:**

Gustavo Parisi,

Silvina Fornasari,

Ana Julia Velez Rueda,

Nicolás Palopoli,

Guillermo Benítez,

Julia Marchetti.

Alexander Monzón,

Marcia Hasenahuer,

Cristian Guisande Donadio,

**Acknowledgments**: To Lao Tzu, Clancy Wiggum, Crazy hatter, Extreme, Pinky and Brain, Whitney Houston, George R.R. Martin, Michael Ende, The Clash for their inspiration.
