# Supplementary material for: Bioinformatics calls the school: Use of smartphones to introduce Python for bioinformatics in high schools
Source: PLoS Comput Biol. 2019 Feb 14;15(2):e1006473. doi: 10.1371/journal.pcbi.1006473 (PMC6375546; doi:10.1371/journal.pcbi.1006473)
Supplement: S2 File — Contest questionnaire and examples of final scripts built by the students answering the required contest questions. (DOCX) [file pcbi.1006473.s002.docx]

**Supplementary Material 2**

**Exercise 1:** Let's say that DNA is nothing more than a code message that must be deciphered or interpreted for the synthesis of proteins. The message is written in a sequence determined by 4 nucleotides represented by the letters A, T, G and C. Within the

cell, the message is transported by another molecule, RNA, very similar to DNA but with U instead of T. In this message, each triplet or group of three letters of the RNA is called a codon, and each amino acid of the proteins is encoded by one or several codons. Thus, by applying this genetic code, it is possible to translate the nucleotide sequence of the RNA into the sequence of amino acids that make up the protein. Let's play, we will be cells for a day! We want to keep a secret very well: a phrase, a message, a magic recipe. Since we are cells and we know the genetic code, we are going to use it to hide the message. The message that they must code is: "If you walk, you will arrive" (Obviously, the comma and the spaces do not have to be encrypted in the genetic code). Given the following table of equivalences that represents the genetic code, propose a program that translates our original message, written with the 20 letters corresponding to amino acids, a new message written with the 64 codons available. For extra points: Most amino acids are encoded by more than one codon. To earn extra points, you can make the program indicate how many different codon sequences can encode the hidden message, and show all those sequences.

**Example 1: ‘Encriptador’.** Answer to question 1 of the 'I Bioinformatics in the Classroom Contest'.

#!/usr/bin/python

# -*- coding: cp1252 -*-

from random import randint

codigo={

"A":["GCU", "GCC", "GCA", "GCG"],

"C":["UGU", "UGC"],

"D":["GAU", "GAC"],

"E":["GAA", "GAG"],

"F":["UUU", "UUC"],

"G":["GGU", "GGC", "GGA", "GGG"],

"H":["CAU", "CAC"],

"I":["AUU", "AUC", "AUA"],

"K":["AAA", "AAG"],

"L":["CUU", "CUC", "CUA", "CUG", "UUA", "UUG"],

"M":["AUG"],

"N":["AAU", "AAC"],

"P":["CCU", "CCC", "CCA", "CCG"],

"Q":["CCA", "CAG"],

"R":["AGA", "AGG", "CGU", "CGC", "CGA", "CGG"],

"S":["AGU", "AGC", "UCU", "UCC", "UCA", "UCG"],

"T":["ACU", "ACC", "ACA", "ACG"],

"V":["GUU", "GUC", "GUA", "GUG"],

"W":["UGG"],

"Y":["UAU", "UAC"],

"stop":["UAA", "UAG", "UGA"] }

frase="Si caminas, llegaras"

print ("Frase 1: '" + frase + "'")

print ("Uno de los genes que podría traducir la frase es:")

for l in frase:

l=l.upper()

if l in codigo.keys():

print (codigo[l][randint(0, len(codigo[l])-1)])

print (codigo["stop"][randint(0, len(codigo["stop"])-1)])

frase="Hola"

print ("Frase más corta: '" + frase + "'")

lista=[]

contador=0

def cantidad(cont):

total=1

gen=[]

for l in frase:

l=l.upper()

if l in codigo.keys():

total*=len(codigo[l])

gen.append(codigo[l][randint(0, len(codigo[l])-1)])

gen.append(codigo["stop"][randint(0,len(codigo["stop"])-1)])

total*=len(codigo["stop"])

if "".join(gen) in lista:

if cont<total:

return cantidad(cont)

elif cont==total:

print ("Estas son las posibles combinaciones de codones con las que cuenta la frase:")

print (lista)

return ("La frase cuenta con " + str(cont) + " posibles genes.")

else:

lista.append("".join(gen))

cont+=1

return (cantidad(cont))

print (cantidad(contador))

**Example 2: ‘Encriptador’.** Answer to question 1 of the 'I Bioinformatics in the Classroom Contest'.

#!/usr/bin/env python

S="UCU"

I="AUU"

C="UGU"

A="GCU"

M="AUG"

N="AAU"

L="CUU"

E="GAA"

G="GGU"

R="CGU"

mensaje = "si caminas llegaras"

mensaje_encriptado=mensaje.replace("s",S).replace("i",I).replace("c",C).replace("a",A).replace("m",M).replace("n",N).replace("l",L).replace("e",E).replace("g",G).replace("r",R)

print(mensaje_encriptado)

**Exercise 2**: Inside the cells, the DNA is stored as a double strand that is held together by A-T and G-C bonds. The following aligned sequences represent four DNA sequences; only the sequence of one of the two threads is shown. Design a program that calculates the % of matings G-C (or C-G) in each double strand of the following sequences:

sec1: TATTTAAATGGATATGGATAATGAACGT

sec2: CACCCAATTGGAATTAATTGGACCGTTA

sec3: GAGGGATTTGGAATTATTGAACGTTACC

sec4: AAAGAAATTGGATAATGAACGTTCGTTA

For extra points: you can make your program calculate the percentage of A-T (or T-A), and say the percentages of each of the nucleotides, in each of the strands, of each of the sequences.

Example 1: CG % calculator. Answer to question 2 of the 'I Bioinformatics in the Classroom Contest'.

#!/usr/bin/env python

sec1="TATTTAAATGGATATGGATAATGAACGT"

sec2="CACCCAATTGGAATTAATTGGACCGTTA"

sec3="GAGGGATTTGGAATTATTGAACGTTACC"

sec4="AAAGAAATTGGATAATGAACGTTCGTTA"

print(len(sec1))

cant_G_sec1=sec1.count('G')

porc_G_sec1=cant_G_sec1*100/len(sec1)

print(porc_G_sec1)

cant_C_sec1=sec1.count("C")

porc_C_sec1=cant_C_sec1*100/len(sec1)

print(porc_C_sec1)

cant_GC_sec1=cant_C_sec1+cant_G_sec1

print(cant_GC_sec1)

porc_GC=cant_GC_sec1*100/len(sec1)

print(porc_GC)

**Example 2:** CG % calculator. Answer to question 2 of the 'I Bioinformatics in the Classroom Contest'.

#!/usr/bin/env python

sec1="TATTTAAATGGATATGGATAATGAACGT"

sec2="CACCCAATTGGAATTAATTGGACCGTTA"

sec3="GAGGGATTTGGAATTATTGAACGTTACC"

sec4="AAAGAAATTGGATAATGAACGTTCGTTA"

def porcentaje(sec):

boh=[]

bah=[]

for n in sec:

boh.append(n)

if n=="G" or n=="C":

bah.append(n)

return len(bah)*100.0/len(boh)

def printear(sec, nro):

return "Los apareamientos de guanina y citocina en la secuencia " + str(nro) + " corresponden al " + str(porcentaje(sec)) + "% del total."

print printear(sec1, 1)

print printear(sec2, 2)

print printear(sec3, 3)

print printear(sec4, 4)

**Exercise 3:** Consider the following alignment of seven DNA sequences, where each column represents equivalent positions in all sequences:

sec1: AAAAA

sec2: TATTT

sec3: CACCC

sec4: GAGGG

sec5: AAAGA

sec6: CGAGC

sec7: GTATG

sec 8: GTATC

Could you design a program that tells us which position (column) is less variable, that is, the one with the highest frequency of equal nucleotides?

For extra points: try to make the program inform, in addition, what is the frequency of each of the four nucleotides per position.

**Example 1:** Answer to question 3 of the 'I Bioinformatics in the Classroom Contest'.

#!/usr/bin/env python

# -*- coding: cp1252 -*-

sec1="AAAAA"

sec2="TATTT"

sec3="CACCC"

sec4="GAGGG"

sec5="AAAGA"

sec6="CGAGC"

sec7="GTATG"

sec8="GTATC"

secs=[sec1, sec2, sec3, sec4, sec5, sec6, sec7, sec8]

def calculo(sec):

letra=0

for n in range(0, len(sec[0])):

bah=[]

for base in sec:

bah.append(base[n])

print bah

def acgt(x):

bas=0

total=0

for l in bah:

total+=1

if l ==x:

bas+=1

return (float(bas)/total*100)

fr_a=acgt("A")

fr_c=acgt("C")

fr_g=acgt("G")

fr_t=acgt("T")

frs=[fr_a, fr_c, fr_g, fr_t]

print (frs)

for f in frs:

if f>50:

print ("Hay menos variabilidad en la posición " + str(n+1))

print (calculo(secs))

Example 2: Answer to question 3 of the 'I Bioinformatics in the Classroom Contest'. #!/usr/bin/env python

sec1="AAAAA"

sec2="TATTT"

sec3="CACCC"

sec4='GAGGG'

sec5='AAAGA'

sec6='CGAGC'

sec7='GTATG'

sec8='GTATC'

col1=[]

col1.append(sec1[0])

col1.append(sec2[0])

col1.append(sec3[0])

col1.append(sec4[0])

col1.append(sec5[0])

col1.append(sec6[0])

col1.append(sec7[0])

col1.append(sec8[0])

print(col1)

porc_A_col1=col1.count('A')*100/len(col1)

print(porc_A_col1)

porc_T_col1=col1.count('T')*100/len(col1)

print(porc_T_col1)

porc_C_col1=col1.count('C')*100/len(col1)

print(porc_C_col1)

porc_G_col1=col1.count('G')*100/len(col1)

print(porc_G_col1)

porc_nuc1=[porc_A_col1,porc_T_col1,porc_C_col1,porc_G_col1]

porc_nuc1.sort(reverse=True)

print(porc_nuc1)

max_porc_col1=(max(porc_nuc1))

print(max_porc_col1)

porc_nuc1.remove(max_porc_col1)

print(max(porc_nuc1))

col2=[]

col2.append(sec1[1])

col2.append(sec2[1])

col2.append(sec3[1])

col2.append(sec4[1])

col2.append(sec5[1])

col2.append(sec6[1])

col2.append(sec7[1])

col2.append(sec8[1])

print(col2)

porc_A_col2=col2.count('A')*100/len(col2)

print(porc_A_col2)

porc_T_col2=col2.count('T')*100/len(col2)

print(porc_T_col2)

porc_C_col2=col2.count('C')*100/len(col2)

print(porc_C_col2)

porc_G_col2=col2.count('G')*100/len(col2)

print(porc_G_col2)

porc_nuc2=[porc_A_col2,porc_T_col2,porc_C_col2,porc_G_col2]

porc_nuc2.sort(reverse=True)

print(porc_nuc2)

max_porc_col2=max(porc_nuc2)

print(max_porc_col2)

porc_nuc2.remove(max_porc_col2)

print(max(porc_nuc2))

col3=[]

col3.append(sec1[2])

col3.append(sec2[2])

col3.append(sec3[2])

col3.append(sec4[2])

col3.append(sec5[2])

col3.append(sec6[2])

col3.append(sec7[2])

col3.append(sec8[2])

print(col3)

porc_A_col3=col3.count('A')*100/len(col3)

print(porc_A_col3)

porc_T_col3=col3.count('T')*100/len(col3)

print(porc_T_col3)

porc_C_col3=col3.count('C')*100/len(col3)

print(porc_C_col3)

porc_G_col3=col3.count('G')*100/len(col3)

print(porc_G_col3)

porc_nuc3=[porc_A_col3,porc_T_col3,porc_C_col3,porc_G_col3]

porc_nuc3.sort(reverse=True)

print(porc_nuc3)

max_porc_col3=max(porc_nuc3)

print(max_porc_col3)

porc_nuc3.remove(max_porc_col3)

print(max(porc_nuc3))

col4=[]

col4.append(sec1[3])

col4.append(sec2[3])

col4.append(sec3[3])

col4.append(sec4[3])

col4.append(sec5[3])

col4.append(sec6[3])

col4.append(sec7[3])

col4.append(sec8[3])

print(col4)

porc_A_col4=col4.count('A')*100/len(col4)

print(porc_A_col4)

porc_T_col4=col4.count('T')*100/len(col4)

print(porc_T_col4)

porc_C_col4=col4.count('C')*100/len(col4)

print(porc_C_col4)

porc_G_col4=col4.count('G')*100/len(col4)

print(porc_G_col4)

porc_nuc4=[porc_A_col4,porc_T_col4,porc_C_col4,porc_G_col4]

porc_nuc4.sort(reverse=True)

print(porc_nuc4)

max_porc_col4=max(porc_nuc4)

print(max_porc_col4)

porc_nuc4.remove(max_porc_col4)

print(max(porc_nuc4))

col5=[]

col5.append(sec1[4])

col5.append(sec2[4])

col5.append(sec3[4])

col5.append(sec4[4])

col5.append(sec5[4])

col5.append(sec6[4])

col5.append(sec7[4])

col5.append(sec8[4])

print(col5)

porc_A_col5=col5.count('A')*100/len(col5)

print(porc_A_col5)

porc_T_col5=col5.count('T')*100/len(col5)

print(porc_T_col5)

porc_C_col5=col5.count('C')*100/len(col5)

print(porc_C_col5)

porc_G_col5=col5.count('G')*100/len(col5)

print(porc_G_col5)

print(porc_G_col4)

porc_nuc5=[porc_A_col5,porc_T_col5,porc_C_col5,porc_G_col5]

porc_nuc5.sort(reverse=True)

print(porc_nuc5)

max_porc_col5=max(porc_nuc5)

print(max_porc_col5)

porc_nuc5.remove(max_porc_col5)

print(max(porc_nuc5))
