# Supplementary material for: Bioinformatics calls the school: Use of smartphones to introduce Python for bioinformatics in high schools
Source: PLoS Comput Biol. 2019 Feb 14;15(2):e1006473. doi: 10.1371/journal.pcbi.1006473 (PMC6375546; doi:10.1371/journal.pcbi.1006473)
Supplement: S3 File — This script was used for testing different platforms—multiple combinations of smartphones models or PCs, with different OSs and Python versions, or online tools. OS, operating system. (PDF) [file pcbi.1006473.s003.pdf]

Demo code for mobile python applications testing. Adapted from C. Soprano, J. Izeta, I. Acosta (Liceo Victor Mercante), originally for 1st Bioinformatics at School contest (Argentina, 2017).

```
#!/usr/bin/env python
# --coding: utf-8--
# sbgeduc_speedtest.py
# Demo code for mobile python applications testing
# Adapted from C. Soprano, J. Izeta, I. Acosta (Liceo Victor Mercante),
# originally for 1st Bioinformatics at School contest (Argentina, 2017)


# import external libraries
from datetime import datetime
import itertools as it

# start time
startTime = datetime.now()

# define genetic code
genetic_code = {
"A":["GCU", "GCC", "GCA", "GCG"],
"C":["UGU", "UGC"],
"D":["GAU", "GAC"],
"E":["GAA", "GAG"],
"F":["UUU", "UUC"],
"G":["GGU", "GGC", "GGA", "GGG"],
"H":["CAU", "CAC"],
"I":["AUU", "AUC", "AUA"],
"K":["AAA", "AAG"],
"L":["CUU", "CUC", "CUA", "CUG", "UUA", "UUG"],
"M":["AUG"],
"N":["AAU", "AAC"],
"P":["CCU", "CCC", "CCA", "CCG"],
"Q":["CCA", "CAG"],
"R":["AGA", "AGG", "CGU", "CGC", "CGA", "CGG"],
"S":["AGU", "AGC", "UCU", "UCC", "UCA", "UCG"],
"T":["ACU", "ACC", "ACA", "ACG"],
"V":["GUU", "GUC", "GUA", "GUG"],
"W":["UGG"],
"Y":["UAU", "UAC"],
".":["UAA", "UAG", "UGA"]
}

# phrase to translate
phrase = "Darwin & Wallace"
print ("Phrase (submitted): '" + phrase + "'")

# get alternative translations
```

```
phrase_elements = list(''.join(letter.upper() for letter in phrase if
letter.upper() in genetic_code))
print ("Phrase (accepted): '" + ''.join(phrase_elements) + "'\n")
alternatives = list(it.product(*(genetic_code[name] for name in
phrase_elements)))

# count alternative translations
counter = len(alternatives)

# print alternative translations
for i,p in enumerate(alternatives):
  print(str(i+1) + ': ' + ''.join(p))

# print number of alternative translations
print('\nThere are '+ str(counter) + ' possible codes')

# print execution time
print('Execution time: '+ str(datetime.now() - startTime))
```
